# Outcomes after suppressive antimicrobial therapy for prosthetic joint infection: a prospective cohort study

**DOI:** 10.1128/aac.01784-24

**Published:** 2025-04-22

**Authors:** Craig Aboltins, Christopher Lemoh, Mani Suleiman, Alex Soriano, Joshua Davis, Laurens Manning

**Affiliations:** 1 Department of Infectious Diseases, Northern Health, Melbourne, Victoria, Australia; 2 Department of Medicine, Northern Health, The University of Melbourne 2281, Melbourne, Victoria, Australia; 3 Department of Medicine at Western Health, The University of Melbourne 2281, Melbourne, Victoria, Australia; 4 Department of Medicine, Monash Health School of Clinical Sciences, Monash University 2541, Melbourne, Victoria, Australia; 5 Research Development and Governance Unit, Northern Health, Melbourne, Victoria, Australia; 6 Research and Industry Engagement, Latrobe University, Melbourne, Victoria, Australia; 7 Department of Infectious Diseases, Hospital Clinic of Barcelona, Barcelona, Spain; 8 IDIBAPS, University of Barcelona 16724, Barcelona, Spain; 9 Menzies School of Health Research, Charles Darwin University 10095, Darwin, Northern Territory, Australia; 10 Department of Infectious Diseases, John Hunter Hospital, Newcastle, New South Wales, Australia; 11 Department of Infectious Diseases, Fiona Stanley Hospital 418838, Murdoch, Western Australia, Australia; 12 Medical School, Faculty of Health and Medical Sciences, The University of Western Australia 172098, Perth, Western Australia, Australia; University of California San Francisco, San Francisco, California, USA

**Keywords:** chronic suppression, antimicrobials, peri-prosthetic joint infection (PJI), suppressive antibiotic therapy (SAT), quality of life score, debridement and implant retention, prospective cohort study

## Abstract

The objective of this study was to describe the use of and outcomes after suppressive antimicrobial therapy (SAT) in a large prospective peri-prosthetic joint infection (PJI) cohort. SAT was defined as antimicrobial therapy continuing beyond 12 months from PJI diagnosis or where there was an early intention for SAT. The primary outcome was “treatment failure” at 24 months, defined as any of (i) clinical evidence of (ii) further surgery for or (iii) death from PJI. Secondary outcomes included quality of life (QOL) scores using Short Form 12 (SF-12) and Oxford hip (OHS) and knee (OKS) scores. SAT was prescribed for 223 of 720 (31.0%) in the cohort. Patients prescribed SAT were more likely to be older, have comorbidities, chronic PJI, higher C-reactive protein, sinus tract, or be treated with debridement and implant retention. The most frequently prescribed antimicrobials for SAT were ciprofloxacin (64 [21%]), amoxicillin (42 [14%]), and rifampicin (35 [12%]). Treatment failure was more common in the SAT group (75/185 [40.1%] vs 85/447 [19.0%]). After propensity score-adjusted analysis, SAT remained associated with higher rates of treatment failure (aOR 2.48, 95% CI [1.66–3.72]). Although 24-month QOL scores were lower in the SAT group, there were similar improvements from baseline in functional joint scores in SAT and non-SAT groups (OHS median interquartile range [IQR] +8.5 [19.0] vs +7.0 [22.0]; *P* = 0.78 and OKS +8.0 [20.0] vs +7.0 [22.0]; *P* = 0.53). SAT use for PJI is common, and in this study, it was not associated with improved outcomes. Identifying patients most likely to benefit from SAT should be explored in carefully designed controlled trials.

## INTRODUCTION

The management of peri-prosthetic joint infection (PJI) is challenging and associated with significant morbidity and cost, particularly where extensive or complex surgery is indicated to attempt to cure the infection. As the number of patients who have undergone total joint replacement increases and as these patients age and develop comorbidities, it becomes increasingly important to develop knowledge around management options for patients for whom extensive surgical intervention would be inappropriate (1, 2). Rather than curing infection, the intention of suppressive antimicrobial therapy (SAT) is to minimize symptoms such as pain and sinus drainage, maintain function, and avoid further surgery in patients who have failed previous treatment or for whom high peri-operative risk precludes curative surgery. SAT has also been used in some patients who have had potentially curative treatment but are deemed to be at high risk of treatment failure due to patient or infection-related factors. The utility of SAT needs to be balanced against the potential downsides of long-term antibiotics, including adverse events, costs, and selection for resistant organisms (3).

Previous studies have described the epidemiology of patients treated with SAT (4). However, there is relatively little high-quality evidence available regarding the efficacy of SAT according to patient factors or the type of PJI, with most previous studies limited by low sample size or a study design being a non-comparative cohort (5). Whilst maintaining function and symptom control are priorities of SAT, few studies have reported the overall functional or quality of life outcomes (3). This study aims to describe the use of SAT in a large prospective cohort of patients with PJI and compare those treated with SAT to patients with PJI treated with alternative surgical and medical therapies, with outcomes including not only control of infection but also mortality, functional, and quality of life outcomes.

## MATERIALS AND METHODS

### Study sites, ethical approval, and participants

This study was a pre-planned secondary analysis of the Prosthetic Joint Infection in Australia and New Zealand (NZ) Observational (PIANO) cohort, which has been previously described in detail (6, 7). PIANO was a prospective, multicenter observational cohort study conducted at 27 hospitals in Australia and NZ. From July 2014 through December 2017, adult patients (>18 years old) with a newly identified large joint PJI were prospectively identified and enrolled after referral from orthopedic and infectious diseases or microbiology teams at each institution. Periprosthetic joint infection was defined according to the 2013 criteria of the Infectious Diseases Society of America, namely the presence of at least one of (i) a sinus tract communicating with the prosthesis; (ii) visible pus around the prosthesis at operation without alternative explanation; (iii) acute inflammation on histopathological examination of periprosthetic tissue (≥5 or more neutrophils per high power field); (iv) two or more pre-operative or intraoperative cultures that yield the same organism; and (v) pure growth of Staphylococcus aureus, beta-hemolytic streptococci or aerobic Gram-negative bacilli from a single synovial fluid or intraoperative tissue/fluid specimen (8). Ethical approvals were obtained from each participating site (ANZCTR12615001357549), and written informed consent was obtained from all participants. All surgical and medical management was determined by individual treating clinicians at each site.

### Data management and statistical analysis

Data were collected by investigators at each site at baseline (PJI diagnosis) and at 90 days, 12, and 24 months after diagnosis into a purpose-built web-based database. Collected data included demographic, clinical, pathological, and microbiological data from a review of the medical record, pathology databases, and direct review of the patient. Information on the type and ongoing prescription of each antimicrobial was collected at each time point. Statistical analysis was conducted using IBM SPSS Statistics (Version 29.0.0.0). Descriptive statistics were used to describe SAT and non-SAT groups. In univariable analyses, χ^2^ or Fisher tests were used for categorical variables. For continuous variables normally distributed, variables were reported as mean and standard deviation (SD), and differences between groups were tested for significance using the Student’s *t*-test. Non-normally distributed continuous variables were reported using the median and interquartile range (IQR), with the Mann-Whitney U test used to test for statistically significant differences across groups. Logistic regression analysis with the single step enter method was used for determining propensity scores and creating regression models predicting the primary outcome.

### Definitions

Early PJI referred to infections occurring within 30 days after the original arthroplasty operation with a symptom duration of ≤7 days. Late-acute PJI refers to infections occurring more than 30 days after arthroplasty with a symptom duration of ≤7 days and no evidence of a sinus overlying the joint. Chronic PJI refers to infections occurring more than 30 days from implantation with more than 30 days of symptoms at diagnosis or the presence of a sinus. The remainder was considered not classifiable. The surgical management approach was categorized at day 90 into the following: (i) two-stage exchange arthroplasty (if at least the first stage had occurred and even if debridement had occurred first), (ii) single-stage exchange arthroplasty (even if debridement had occurred first), (iii) excision arthroplasty (even if debridement had occurred first and there was no plan for prosthesis reimplantation), (iv) debridement and implant retention (DAIR), and (v) no surgical intervention (7, 9).

SAT was defined as antibiotic treatment given to patients for PJI, which continued beyond 12 months after diagnosis or after a period of less than 12 months where there was a specified intent for SAT.

The primary outcome was treatment failure at 24 months from the time of diagnosis defined as any of (i) the requirement for further surgery additional to the initial surgical management approach at day 90 to control the infection (debridement, prosthesis exchange, or removal/amputation), (ii) ongoing or the development of clinical or microbiological evidence of infection, or (iii) death due to PJI or its treatment. The cause of death (related to PJI or not) was, where possible, determined by the treating clinician/site study lead clinician. Secondary outcomes were the retention of the “destination prosthesis,” which was defined as the prosthesis remaining *in situ* after the initial surgical management approach. Oxford hip and knee scores (OHS and OKS) were captured at baseline and at 24 months. A Short Form-12 (SF-12 v2.0) was also completed at each time point. Individual physical component and mental component scores (PCS and MCS, respectively) were scored against age-adjusted population normative values for the Australian population (9).

To attempt to control for confounding between groups receiving and not receiving SAT, we applied inverse probability of treatment weighting based on propensity scoring that included age, gender, joint (knee vs hip), chronic renal failure, cirrhosis, ischaemic heart disease, rheumatoid arthritis, baseline C-reactive protein (CRP), the presence of positive blood cultures at baseline, clinical type of infection (early vs others), and the presence of *Staphylococcus aureus*. After weighting, regression analysis with a robust estimation was performed to increase the precision of the comparison of outcomes between groups. Results were stratified by the clinical type of infection, presentation with a sinus tract, the presence of ongoing symptoms at day 90, management with DAIR, and infecting microorganism.

## RESULTS

**Fig 1:**
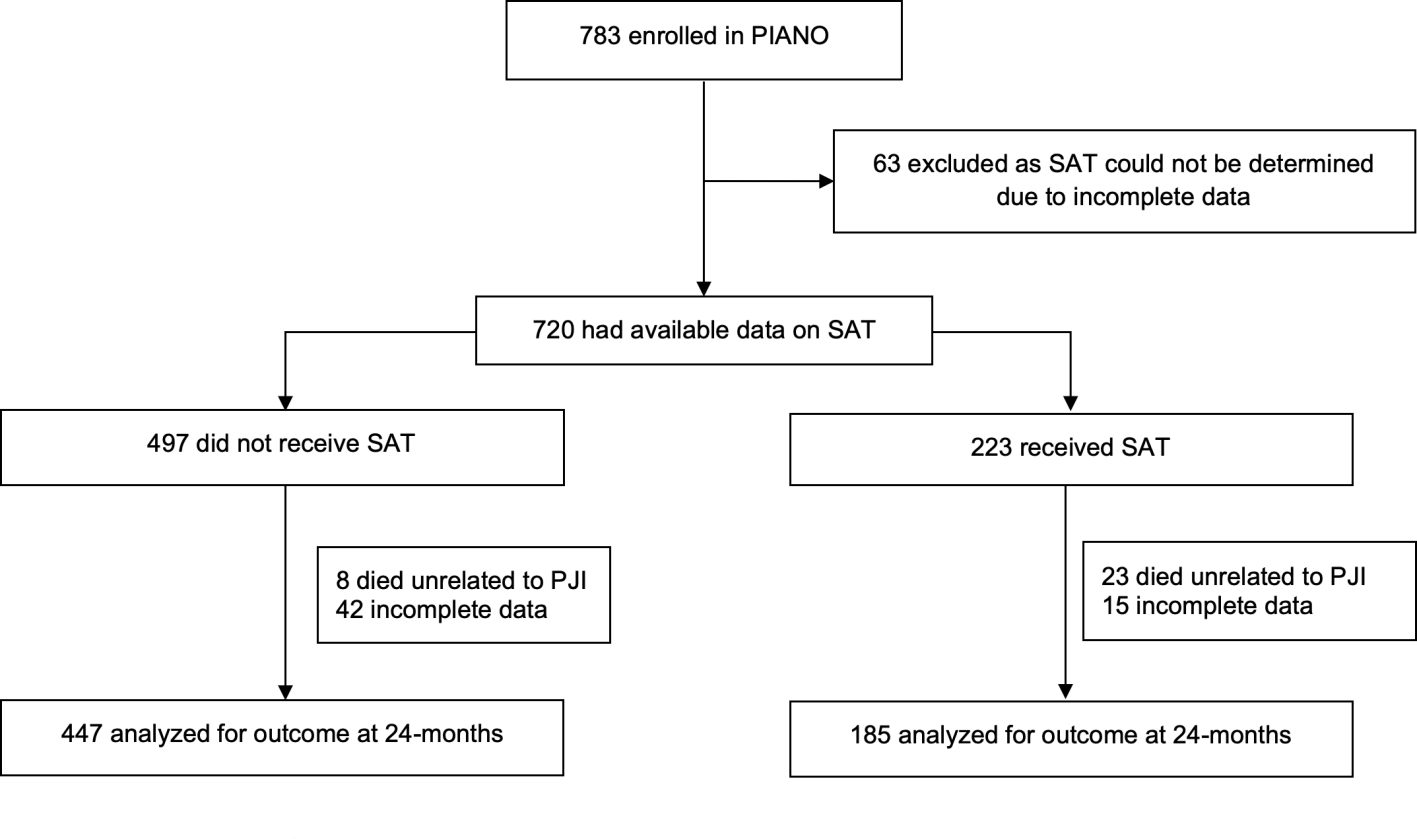
Flow diagram of patients.

Of the 783 patients originally included in the PIANO cohort, information was available to determine if SAT had been prescribed in 720. Of these, 223 (31.0%) were prescribed SAT, and 497 (69%) were not. Of those prescribed SAT, the intention to manage with SAT had been identified by the day 90 review in 78 (34.9%). Primary outcome data were available for analysis for 632 at 24 months (Fig. 1). The baseline characteristics of those with primary outcome data at 24 months were similar to those without primary outcome data (*n* = 151), with similar mean age of those with outcome data (68.9 years vs 68.9 years), male sex (365 [57.8%] vs 85 [58.3%]), having at least one comorbidity (296 [46.8%] vs 78 [51.7%]), early PJI (157 [25.1%] vs 40 [26.8%]) and having isolated *Staphylococcus aureus* (259 [41.0%] vs 60 [39.7%]), respectively.

At 24 months, more patients prescribed SAT died from non-PJI causes (23/223) than patients not prescribed SAT (8/497; *P* < 0.00001).

Baseline characteristics of all patients according to the use of SAT are shown in Table 1. Patients prescribed SAT were older and were more likely to have comorbidities, especially cardiac failure, renal failure, and malignancy. They were more likely to have presented with chronic PJI compared with acute PJI, with a longer duration of symptoms, a sinus tract, and a higher baseline CRP. The microbiological aetiology was not significantly different between the groups. Surgical treatment was different between the groups, with those prescribed SAT more likely to have had a DAIR procedure than patients not prescribed SAT. SAT was prescribed for 28/134 (20.9%) of patients with early PJI treated with DAIR, 75/211 (35.5%) with late acute PJI treated with DAIR, and 16/64 (25.0%) with chronic PJI treated with two-stage exchange. Of 11 patients without any surgical intervention, SAT was prescribed in 9 (82%).

**TABLE 1:** Baseline characteristics of patients not prescribed and prescribed SAT^*c*^

	Non-SAT group (*n* = 497)	SAT group (*n* = 223)	*P*-value
Age in years^*a*^	68.0 (9.6)	71.6 (12.0)	<0.001
Male	298 (60)	119 (53)	0.10
BMI^*b*^ kg/m^2^	31.1 (9.3)	31.4 (11.0)	0.41
Any comorbidity	215 (43)	128 (57)	<0.001
Cardiac failure	17 (3)	23 (11)	<0.001
Chronic renal failure	30 (6)	29 (13)	0.003
Diabetes mellitus	107 (22)	51 (23)	0.70
Immunosuppression	27 (5)	15 (7)	0.49
Ischaemic heart disease	74 (15)	45 (20)	0.08
Malignancy	11 (2)	20 (9)	<0.001
Index joint			0.35
Ankle	2 (0)	0 (0)	
Elbow	4 (1)	1 (0)	
Hip	210 (42)	84 (38)	
Knee	262 (53)	133 (60)	
Shoulder	19 (4)	5 (2)	
Indication for implant			<0.001
Primary	424 (85)	154 (69)	
Infection	21 (4)	10 (4)	
Other/unknown	52 (10)	59 (26)	
			
Duration of symptoms in days^*b*^	4 (11)	5 (13)	0.04
Fever >38°C	192 (39)	95 (43)	0.32
Local inflammation	389 (68)	184 (83)	0.23
Sinus	73 (15)	50 (22)	0.01
Septic shock	19 (4)	14 (6)	0.18
White cell count^*b*^ × 10^9^/L	11.9 (7.1)	11.6 (7.1)	0.52
Category of PJI			<0.001
Early	145 (29)	36 (16)	
Late acute	203 (41)	94 (42)	
Chronic	96 (19)	56 (25)	
Not classifiable	53 (11)	37 (17)	
			
Serum Cr^*b*^ μmol/L	85 (40)	81 (53)	0.72
Bilirubin^*b*^ μmol/L	10 (9)	9 (9)	0.73
C-reactive protein^*b*^ mg/L	173 (219)	190 (201)	0.04
Causative organism			
*Staphylococcus aureus*	195 (40)	98 (44)	0.25
Coagulase negative staphylococcus	112 (23)	39 (18)	0.14
Streptococcus	108 (22)	51 (23)	0.77
Enterococcus	29 (6)	17 (8)	0.40
Cutibacterium	21 (4)	9 (4)	1.00
Gram negative bacillus	65 (13)	48 (22)	0.005
Polymicrobial	113 (26)	42 (20)	0.17
Culture-negative	54 (11)	15 (7)	0.10
			
Clinical evidence of ongoing infection at day 90	60 (12)	54 (25)	<0.001
Surgical management at day 90			<0.001
DAIR	298 (60)	156 (70)	
Excision arthroplasty	21 (4)	4 (2)	
1-stage revision	30 (6)	13 (6)	
2-stage revision	140 (28)	37 (17)	
No surgery	2 (0)	9 (4)	
Duration of iv antibiotics by day 90 (days)^*b*^	42.0 (12)	42.0 (18.6)	0.67

^*a*^ Mean (SD).

^*b*^ Median (IQR).

^*c*^ Values are numbers (percentages) unless otherwise indicated.

**Fig 2:**
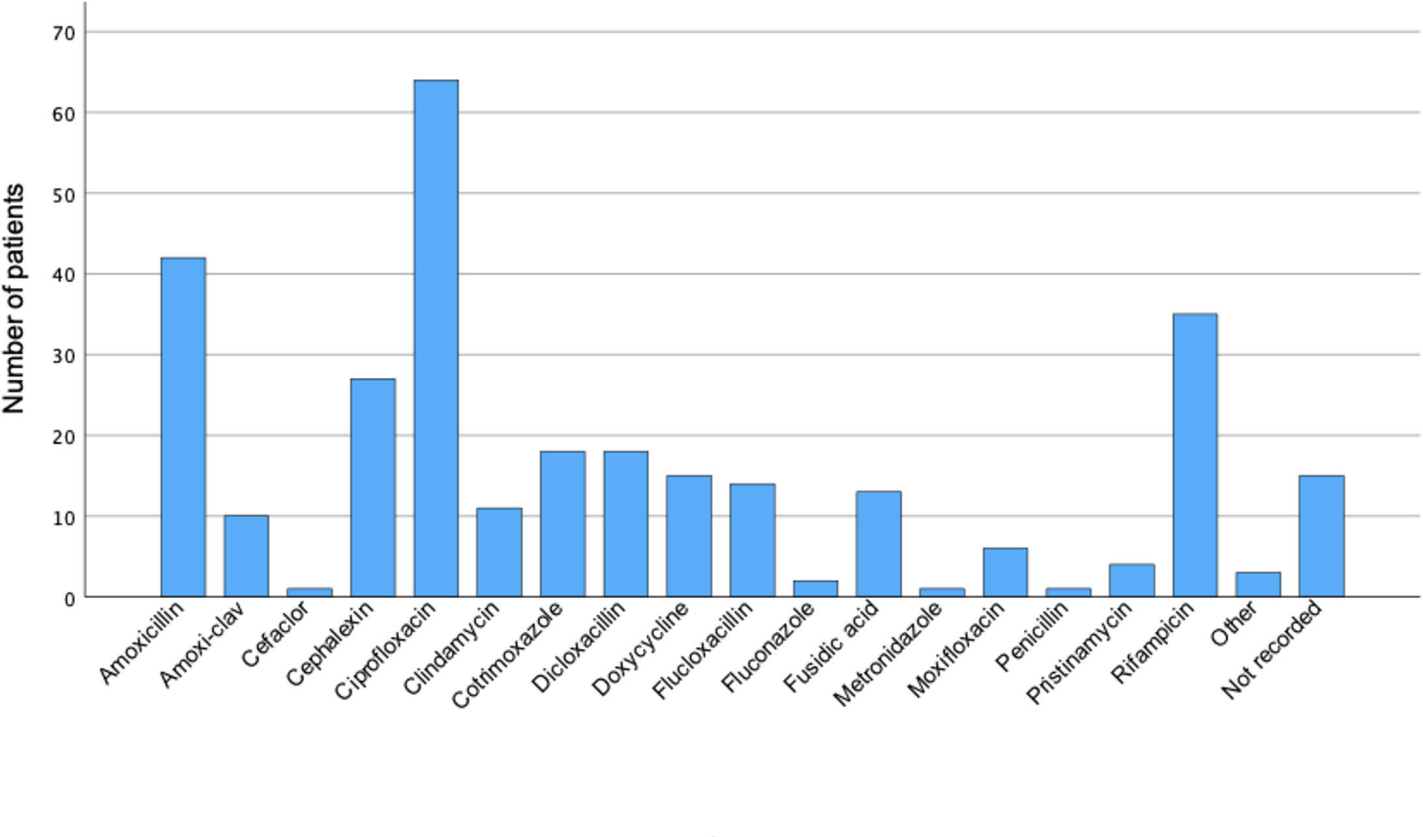
Antimicrobials used for SAT. Amoxi-clav, amoxicillin-clavulanic acid.

The antimicrobials prescribed to patients for SAT are shown in Fig. 2. A total of 161 patients were concurrently prescribed one antimicrobial, 50 patients were prescribed two, 9 patients were prescribed three, and 3 patients were prescribed four antimicrobials. The most frequently prescribed antimicrobials were ciprofloxacin (64 [21%]), amoxicillin (42 [14%]), rifampicin (35 [12%]), and cephalexin (27 [9%]). The median duration of all antibiotics in those who did not receive SAT was 85 days (IQR 128). Six patients receiving long-term antibiotics with a spectrum of activity unlikely to treat their PJI and for an indication other than the PJI were included in the group not prescribed SAT. Only 21 patients prescribed SAT ceased antimicrobials between 12 and 24 months post-diagnosis.

The characteristics of patients experiencing treatment failure at 24 months as well as the criteria for failure according to SAT, are shown in Table 2. Of the 160 patients who experienced treatment failure, 137 (85.6%) failed within 12 months, and 23 failed between 12 and 24 months. No patients died from PJI after 12 months. Higher proportions of patients prescribed SAT experienced overall treatment failure, new clinical evidence of infection, and further surgery for PJI at 24 months but there was no significant difference between groups in death due to PJI. In patients treated with DAIR, a higher proportion of patients experienced overall treatment failure in those prescribed SAT compared with those not prescribed SAT (42/142 [33.9%] vs 52/263 [19.8%]; *P* = 0.003). In patients who had resolution of their symptoms at 3 months, those prescribed SAT were more likely to experience treatment failure by 24 months than those who were not prescribed SAT (50/137 [36.5%] vs 53/384 [13.8%]; *P* ≤ 0.0001). At 24 months, 381 (87.4%) in the non-SAT group and 140 (80.9%) of patients prescribed SAT still retained their destination prosthesis (*P* = 0.055).

**TABLE 2:** Treatment failure and causes at 24 months according to SAT

	Total (%)	Non-SAT group (%); *n* = 447	SAT group (%)*; n* = 185	*P*-value (non-SAT vs SAT groups)
Treatment failure^*a*^	160 (25.3)	85 (19.0)	75 (40.5)	<0.001
-Death due to PJI	20 (3.2)	13 (2.9)	7 (3.8)	0.62
-New clinical evidence of infection	53 (8.4)	20 (4.5)	33 (17.8)	<0.001
-Further surgery for infection	126 (19.4)	72 (16.1)	54 (29.1)	<0.001

^*a*^ Subjects could have met more than one criterion for the definition of treatment failure.

Fifteen patients who previously had resolution of PJI symptoms developed treatment failure within 4 weeks of ceasing an antibiotic course of longer than 3 months, with 12 failures in the non-SAT group and three failing in the SAT group. Of patients who failed between 12 and 24 months, those in the SAT group were not more likely to fail than those in the non-SAT group (14/75 vs 9/85; *P* = 0.18). Only 2 of 21 (9.5%) patients in the SAT group who ceased antimicrobials between 12 and 24 months subsequently developed treatment failure.

The numbers of patients experiencing treatment failure for different SAT antimicrobials prescribed for different organisms can be seen in Table S1. No antimicrobial was associated with treatment failure for any group of organisms.

After adjustment for propensity score, the prescription of SAT was associated with higher treatment failure, and there was no evidence for lower treatment failure across subgroups according to clinical presentation, infecting organism, and surgical treatment (Table 3).

**TABLE 3:** SAT association with failure of treatment in all PJI patients and in subgroups according to clinical presentation, surgical strategy, and organism

Group	aOR^*a*^	95% CI
All PJI patients	2.48	1.66–3.72
Chronic PJI	1.55	0.70–3.47
Late-acute PJI	2.12	1.18–3.81
Presentation with sinus tract	1.96	0.78–5.07
Ongoing symptoms at day 90	1.13	0.49–2.62
DAIR	1.85	1.11–3.10
*Staphylococcus aureus*	1.84	1.02–3.31

^*a*^ Logistic regression after inverse probability of treatment weighting.

Amongst those prescribed SAT, a higher baseline CRP (median 223 mg/L in those who failed vs 175 mg/L, *P* = 0.004) and younger age (mean 67.7 in those who failed vs 73.4 years, *P* = 0.002) were independently associated with treatment failure, whereas culture-negative PJI was associated with a lower risk of failure (Table S2).

Paired baseline and 24-month OHS were available for 201 patients, OKS for 284 patients, and SF-12 PCS/MCS for 476 patients. Table 4 summarizes these scores. At 24 months, median OKS and OHS were lower in those prescribed SAT; however, there were no differences in the change between baseline and 24-month OHS or OKS between the groups. The median SF-12 PCS amongst patients prescribed SAT was lower at 24 months, and there was a significantly smaller increase from baseline. The median SF-12 MCS score was lower at baseline for those prescribed SAT; however, they had similar improvements in 24-month scores to those not prescribed SAT.

**TABLE 4:** Oxford hip and knee scores and SF-12 scores according to suppressive antimicrobial therapy^*a*^^,^^*b*^

Score	No SAT	SAT	*P*-value
Oxford hip score			
Baseline	26.0 (21)	22.0 (22)	0.18
24 months	40.0 (16)	34.0 (20)	0.017
Difference	7.0 (22)	8.5 (19)	0.78
Oxford knee score			
Baseline	24.0 (20)	25.0 (19)	0.98
24 months	37.0 (14)	29.0 (17)	<0.001
Difference	7.0 (22)	8.0 (20)	0.53
SF-12 PCS			
Baseline	37.9 (16)	36.4 (15)	0.22
24 months	44.5 (17)	38.1 (15)	<0.001
Difference	4.7 (17)	0.5 (16)	0.008
SF-12 MCS			
Baseline	49.9 (18)	46.5 (20)	0.04
24 months	53.8 (15)	52.2 (18)	0.48
Difference	3.8 (19)	5.5 (17)	0.14

^*a*^ All figures are median score (IQR).

^*b*^ SF-12, 12-item Short Form survey; PCS, physical component summary; MCS, mental component summary.

## DISCUSSION

In this study, which includes patients from a large, prospective, multicenter cohort of patients with PJI, SAT use was common, being prescribed for 31% of patients. Other contemporary studies show that SAT was chosen for between only 8% and 15% of PJI patients; however, its use has been described for up to 36.5% of patients over the age of 80, and older studies describe it being used commonly for patients undergoing DAIR (10–14). Patients in this study were more likely to receive SAT if there were features present that have previously been associated with PJI treatment failure, such as older age, medical comorbidities, presentation with a sinus tract, and a higher baseline CRP (15, 16); however, SAT was still used in a significant minority of patients who would usually be considered to have curable infections, such as patients with early PJI treated with DAIR or those with chronic PJI treated with two-stage exchange.

For patients treated with SAT in this study, 59.5% did not experience treatment failure, which is a similar rate to most recently published cohorts where between 56% and 83% of patients were deemed to have had successful treatment. However, comparisons between studies are difficult, given differing definitions of success and inclusion of patients with different clinical infection types and surgical strategies (3, 5, 14). Our study, relative to others, had a higher proportion of patients with late-acute rather than early or chronic PJI, did not exclude patients where antimicrobials were started due to ongoing wound symptoms, and included patients managed with a variety of surgical strategies. Previous studies examining SAT have used definitions of SAT of antimicrobial use for 3 or 6 months; however, known variation in clinical practice can mean that patients on longer defined durations of antimicrobials with curative intent could be misclassified (17). In our study, the definition used of 12 months of antimicrobials may more accurately identify patients where the intention was for long-term suppression rather than curative intent. An important strength of this study is that it included patients where there was an intention to prescribe long-term antibiotics but who experienced treatment failure prior to a given duration of antimicrobials being taken, a group of patients not included in many previous studies examining SAT.

Despite the prospective collection of data and adjusting for multiple patient and infection-related factors that have previously been associated with the failure of PJI treatment in the propensity scoring, SAT was associated with higher odds of treatment failure. Given that there is unlikely a plausible causative association between SAT and an increased risk of treatment failure, this highlights the difficulty in controlling for selection bias for this particular intervention, with patients likely being prescribed SAT because they have been judged by their clinicians to be at high risk of failure. Nonetheless, the finding of a lack of association of SAT with improved outcome is important itself. This outcome was also seen after stratification for chronic or late-acute PJI, presentation with a sinus tract, treatment with DAIR, ongoing symptoms after initial treatment, and staphylococcal PJI, which are important subgroups of patients for which SAT is often considered to be indicated. Our results contrast with one of the two previous matched cohort studies on this topic (12) and confirm the results of the other (14), noting that both of these studies focused only on patients undergoing DAIR.

Amongst patients prescribed SAT in our study, a factor associated with a higher treatment failure was elevated CRP at presentation, a feature associated with failure of PJI treatment in general and likely reflecting the burden of infection on presentation. The association of failure of SAT with younger age contrasts with findings from most cohorts of PJI patients, where older age is a widely recognized risk factor. However, it has been seen previously for patients treated with SAT (4) and may relate to only young patients with the worst prognosis for treatment outcome being selected for SAT. Despite relatively few patients experiencing culture-negative PJI in this cohort, it was associated with a lower rate of treatment failure. Although the cause of this association isn’t clear, it is conceivable that organisms suppressed in operative culture specimens by brief pre-operative courses of antibiotics may be more easily suppressed in the longer term (18).

The most frequently used antimicrobial in this cohort was ciprofloxacin, which has been relatively infrequently used in previous studies. This is potentially related to both regional differences in sensitivity patterns in Gram-negative infections and the use of ciprofloxacin in combination with rifampicin for staphylococcal infections in Australia and New Zealand, where levofloxacin is not marketed. The oral beta-lactams cephalexin, di-/flu-cloxacillin, and amoxicillin were also commonly used, likely reflecting the priority to use agents that are relatively well tolerated and accessible through pharmaceutical benefits schemes. Our study, like previous studies, was not able to demonstrate that a particular antimicrobial was associated with a better or worse outcome for any of the causative organisms, suggesting that if SAT is attempted, antimicrobial choice would be reasonably guided by sensitivity testing, tolerance, and convenience.

Despite an increased rate of failing treatment, patients prescribed SAT had similar improvements in OHS and OKS over the study period to those not prescribed SAT. Given that two of the objectives of SAT are the improvement of joint symptoms and function, which the OHS and OKS focus on, it is possible that SAT may have had a role in improving these patient-centred factors in a group of patients with higher rates of treatment failure as defined by clinicians. Improvement in local joint symptoms despite the failure of SAT has also been seen in a previously published large SAT cohort (4). In our study, there was less improvement in the SAT group in overall physical health as estimated by the SF-12 PCS score, which, although potentially related to receiving SAT, may also be related to the higher rate of comorbidities in this group (19). SF-12 MCS scores improved to a similar extent in patients prescribed SAT to those not, suggesting that neither receiving chronic antibiotics nor the higher rate of treatment failure impacted patients’ mental health significantly.

This study has important limitations. Given the lack of non-random allocation of treatment, there was an inability to completely control for bias in determining the efficacy of SAT. However, the prospective collection of and controlling for multiple factors known to be associated with PJI treatment failure, as well as identifying those where the early intention was for SAT, was designed to minimize such bias. Importantly, information was not collected regarding the complications not only of SAT but also surgical treatments, both of which are important considerations in determining management approaches. The follow-up period of 2 years from the diagnosis of PJI in this study may not be long enough to capture all treatment failures, and this may not have been balanced across the SAT and non-SAT groups, with previous evidence showing that although a majority of failures of PJI treatment occur within this period, not all do (4, 12).

The findings from this large multi-centre PJI cohort have important implications for the current treatment of and future research into PJI management. The frequent use of SAT, the lack of evidence for benefit in controlling infection in multiple subgroups of patients for whom it is often considered an indication, and the potential for improvement in joint function despite a higher treatment failure rate highlight the importance of clarifying this issue. Further prospective controlled studies are needed, focusing on better defining subgroups of patients who benefit from SAT and that include patient-centred outcomes.

## SUPPLEMENTAL MATERIAL

Supplemental material aac.01784-24-s0001.docx Tables S1 and S2.

ASM does not own the copyrights to Supplemental Material that may be linked to, or accessed through, an article. The authors have granted ASM a non-exclusive, world-wide license to publish the Supplemental Material files. Please contact the corresponding author directly for reuse.
