# Supplementary material for: Outcomes after suppressive antimicrobial therapy for prosthetic joint infection: a prospective cohort study
Source: Antimicrob Agents Chemother. 2025 Apr 22;69(6):e01784-24. doi: 10.1128/aac.01784-24 (PMC12135506; doi:10.1128/aac.01784-24)
Supplement: Supplemental material — Tables S1 and S2. [file aac.01784-24-s0001.docx]

**Supplementary material**

**Table S1** Treatment failure at 24 months for patients prescribed SAT according to antibiotic used for different organisms

| **Organism(n)** | **n** | **Prescribed SAT (%)** | **Antimicrobial(n)^a^** | **n** | **Treatment failure (%)** | ***P-*value** |
| --- | --- | --- | --- | --- | --- | --- |
| *Staphylococcus aureus* | 259 | 83 (32.0) | Cephalexin | 19 | 8 (42.2) | 0.27 |
|  |  |  | Cotrimoxazole | 8 | 5 (62.5) | 0.25 |
|  |  |  | Di-/flu-cloxacillin | 24 | 6 (25.0) | 0.07 |
|  |  |  | Rifampicin^b^ | 14 | 6 (42.9) | 1.0 |
| Coagulase-negative staphylococci | 139 | 36 (25.9) | Cotrimoxazole | 5 | 1 (20) | 0.34 |
|  |  |  | Doxycycline | 9 | 6 (66.7) | 0.27 |
|  |  |  | Rifampicin^b^ | 9 | 5 (55.6) | 0.71 |
| Streptococci | 143 | 46 (32.2) | Amoxicillin^c^ | 27 | 7 (25.9) | 0.75 |
|  |  |  | Clindamycin | 4 | 2 (50) | 0.57 |
|  |  |  | Cotrimoxazole | 4 | 0 | 0.31 |
|  |  |  | Moxifloxacin | 4 | 1 (25) | 1.0 |
| Gram-negative bacillus | 92 | 37 (40.2) | Amoxicillin^c^ | 10 | 5 (50) | 0.28 |
|  |  |  | Ciprofloxacin | 22 | 9 (40.9) | 0.51 |
|  |  |  | Cotrimoxazole | 4 | 0 | 0.28 |
| Enterococci | 41 | 15 (36.6) | Amoxicillin^c^ | 9 | 4 (44.4) | 1.0 |

^a^ Only antimicrobials prescribed for 4 or more patients for each organism are shown

^b^ Used in combination with ciprofloxacin or fusidic acid

^c^ Includes amoxicillin, amoxicillin-clavulanic acid and phenoxymethylpenicillin

**Table S2** Factors associated with treatment failure at 24 months in patients prescribed SAT

| **Variable** | **OR** | **95% CI** | ***P*-value** |
| --- | --- | --- | --- |
| Age | 0.96 | 0.94-0.99 | 0.002 |
| Male sex | 1.03 | 0.57-1.85 | 0.94 |
| Prosthesis location (knee) | 1.53 | 0.83-2.83 | 0.18 |
| Revision as indication for primary surgery | 1.01 | 0.53-1.93 | 0.97 |
| Positive blood cultures | 1.52 | 0.79-2.93 | 0.21 |
| Baseline serum Creatinine | 1.00 | 0.99-1.01 | 0.19 |
| Baseline C-reactive protein | 1.003 | 1.001-1.006 | 0.004 |
| Ischaemic heart disease | 1.40 | 0.68-2.92 | 0.36 |
| Malignancy | 3.16 | 0.92-10.92 | 0.07 |
| Any comorbidity | 1.47 | 0.81-2.66 | 0.20 |
| *Staphylococcus aureus* | 1.13 | 0.63-2.04 | 0.68 |
| Gram-negative bacillus | 1.32 | 0.64-2.73 | 0.46 |
| Culture-negative | 0.12 | 0.02-0.96 | 0.046 |
| Early PJI | 0.70 | 0.30-1.66 | 0.42 |
